# Neuroinflammation associated with scrub typhus and spotted fever group rickettsioses

**DOI:** 10.1371/journal.pntd.0008675

**Published:** 2020-10-22

**Authors:** James Fisher, Galen Card, Lynn Soong

**Affiliations:** 1 Department of Microbiology and Immunology, University of Texas Medical Branch, Galveston, Texas, United States of America; 2 Department of Pathology, University of Texas Medical Branch, Galveston, Texas, United States of America; 3 Institute for Human Infections and Immunity, University of Texas Medical Branch, Galveston, Texas, United States of America; University of Oxford, UNITED KINGDOM

## Abstract

Scrub typhus and spotted fever rickettsioses (SFR) are understudied, vector-borne diseases of global significance. Over 1 billion individuals are at risk for scrub typhus alone in an endemic region, spanning across eastern and southern Asia to Northern Australia. While highly treatable, diagnostic challenges make timely antibiotic intervention difficult for these diseases. Delayed therapy may lead to severe outcomes affecting multiple organs, including the central nervous system (CNS), where infection and associated neuroinflammation may be lethal or lead to lasting sequelae. Meningitis and encephalitis are prevalent in both scrub typhus and SFR. Additionally, case reports detailing focal neurological deficits have come to light, with attention to both acute and chronic sequelae of infection. Despite the increasing number of clinical reports outlining neurologic consequences of these diseases, relatively little research has examined underlying mechanisms of neuroinflammation. Animal models of scrub typhus have identified cerebral T-cell infiltration and vascular damage associated with endothelial infection and neuropathogenesis. Differential gene expression analysis of brain tissues during murine scrub typhus have revealed selective increases in CXCR3 ligands, proinflammatory and type-1 cytokines and chemokines, and cytotoxicity molecules, as well as alterations in the complement pathway. In SFR, microglial expansion and macrophage infiltration contribute to neurological disease progression. This narrative Review highlights clinical neurologic features of scrub typhus and SFR and evaluates our current understanding of basic research into neuroinflammation for both diseases in animal models. Further investigation into key mediators of neuropathogenesis may yield prognostic markers and treatment regimens for severe patients.

## Introduction

Scrub typhus and spotted fever rickettsioses (SFR) are arthropod-borne infectious diseases of globally widening impact. The etiological agent of scrub typhus, *Orientia tsutsugamushi*, is transmitted via the bite of the *Leptotrombidium* mite (chigger) and remains endemic to a region spanning most of Asia to Northern Australia, termed the tsutsugamushi triangle [1]. More than one billion people in the tsutsugamushi triangle are at risk of infection, with an estimated one million new cases per year [2]. Recently, reports of scrub typhus in South America and Africa emerged, raising concerns that the disease is either underappreciated or expanding in those regions [3,4]. *O*. *tsutsugamushi* is an obligately intracellular bacterium that targets endothelial cells and phagocytes for replication [1]. Accordingly, disease pathogenesis predominates in highly vascularized organs (lung, liver, brain, etc.), manifesting as interstitial pneumonia, liver damage, and meningoencephalitis [1]. If left untreated, scrub typhus may lead to multiorgan failure, with fatality rates ranging from 0% to 70%, with a median of 6% [1,5,6].

The etiological agents of SFR, on the other hand, are found on all continents except Antarctica and are transmitted via the bite of multiple tick and mite genera [7]. SFR encompass many diseases, the most well-known in the Americas being Rocky Mountain spotted fever (*R*. *rickettsii*), Mediterranean spotted fever (*R*. *conorii*), and rickettsialpox (*R*. *akari*) [7]. Like *Orientia*, *Rickettsia* species are obligately intracellular pathogens with endothelial tropism [8]. Therefore, manifestations often mirror those of scrub typhus, with nonspecific symptoms including rash, malaise, fever, headache, and cough [9]. Severe manifestations may include interstitial pneumonia, acute respiratory distress syndrome, and encephalitis with fatality rates ranging from 15% to 65% [8].

Neurological manifestations and sequelae of scrub typhus and SFR have been overlooked. Numerous reports indicate that both *Orientia* and *Rickettsia* are significant causes of encephalitis and meningitis in the Asia-Pacific region [10–13]. *O*. *tsutsugamushi* accounts for 17.9% of all bacterial central nervous system (CNS) infections in Laos and up to 25% of all encephalitic infections in India [10,13]. *Rickettsia spp*. account for 11% of all CNS infections in Laos, with a case fatality rate of 27% [14]. Yet, little is known about the pathogenesis associated with *Orientia* and *Rickettsia* infections, and even less is known about neuroinflammation associated with these infections. Many current reviews on rickettsial diseases are focused on the genus *Rickettsia* but either omit or briefly mention *O*. *tsutsugamushi*. Here, we compare clinical symptoms, signs, and sequelae of SFR and scrub typhus as well as the molecular pathogenesis of both diseases, highlighting new evidence from related mouse models.

## Methods

### Databases, search strategy, and study selection

Articles for scrub typhus and SFR were identified through scouring relevant publications from electronic sources. Searching was performed via Ovid-Medline and Pubmed-Medline. For scrub typhus, the electronic databases were parsed using “scrub typhus” or “O. tsutsugamushi,” “scrub typhus or O. tsutsugamushi and neurology,” “scrub typhus or O. tsutsugamushi and CNS.” For SFR, the terms “Rickettsia,” “Rickettsia and neurology,” and “Rickettsia and CNS” were utilized. JRF and GEC reviewed abstracts generated by the search for relevance and, unless a seminal publication, only included publications from the recent 5 years. Case reports detailing typhus group rickettsia and scrub typhus super and/or coinfection were also excluded.

## Clinical neurological profiles of scrub typhus and SFR

### Common acute neurological manifestations of scrub typhus and SFR

The most common constellation of neurological signs and symptoms in acute scrub typhus and SFR are those of meningitis, encephalitis, or a combination of the two, termed meningoencephalitis. Definitions for meningitis and encephalitis follow the World Health Organization 2003 guidelines [15]. Meningitis is defined as acute onset of fever (more than 38°C) with at least one of the following: neck stiffness, meningeal signs, or altered consciousness. Acute encephalitis is defined as fever accompanied with either a change in mental status or seizure. Patients with either scrub typhus or SFR tend to exhibit classical signs and symptoms associated with bacterial or viral CNS infection. These are serious, life-threatening signs that require hospitalization. As such, the reviewed studies rely heavily on inpatient data. Fever is present in 100% of meningitis cases, and headache has been identified to occur in 89% of scrub typhus and 96% of SFR meningitis patients [14]. Vomiting is common, occurring in anywhere from 60% to 73% of scrub typhus and about 40% of SFR meningitis cases [14,16]. Meningeal signs (neck stiffness, etc) occur in 49% to 67% of scrub typhus and 50% of SFR patients, respectively [14,16]. Only one report of meningismus in scrub typhus exists [17]. The intracellular nature of *O*. *tsutsugamushi* and *Rickettsia spp*. yields cerebrospinal fluid (CSF) studies nearly identical to aseptic meningitis and similar to tubercular meningitis, complicating efforts to initiate appropriate antibiotic treatment [17,18]. However, CSF studies in scrub typhus have revealed an increased white cell count and 2-fold increased neutrophil-to-lymphocyte ratio compared with *Rickettsia spp*. infection [14]. While the importance of CSF findings during diagnosis is unclear, these reports indicate unrecognized differences in the neuroimmune responses between the two infections.

Acute encephalitis syndrome occurs in 37% to 48% of scrub typhus and 57% of SFR patients experiencing neurological disease [14,18]. Patients present with seizures, altered sensorium, and various other signs, including spastic dysarthria and limb ataxia [18]. Altered sensorium with CSF pleocytosis has been used as criteria for encephalitis in patients diagnosed with scrub typhus [16,18]. Electroencephalogram studies of patients with scrub typhus encephalitis reveal predominant diffuse slowing which may be indistinguishable from virus- or parasite-related encephalitic infections common to endemic regions (e.g., Japanese encephalitis virus, chikungunya virus, or *Plasmodium falciparum*) [18]. Despite the dramatic presentation of patients with scrub typhus meningoencephalitis, these patients tend to have significant improvement of symptoms with doxycycline or minocycline treatment, a finding not as common during neurological complications of rickettsial infection [18,19]. Additionally, those with scrub typhus experiencing seizures also respond well to antiepileptic medications [18].

One study has examined differences in *O*. *tsutsugamushi* and rickettsial acute neuroinflammation utilizing biomarkers, focusing on blood–brain barrier (BBB) function and CSF in 66 patients with CNS infections in Laos [20]. Patients were included as part of a hospital-based analysis of CNS infections for bacterial meningitis, *Mycobacterium tuberculosis* meningitis, Japanese B encephalitis virus, and rickettsial infections, including *O*. *tsutsugamushi*, *R*. *typhi*, and “other” *Rickettsia* species. To assess BBB leakiness, the CSF to plasma albumin ratio (albumin index) was analyzed, in conjunction with other biomarkers, including glial fibrillary acidic protein (astrocyte marker), S100b (astroglia cell marker), neuron-specific enolase, and total tau protein (axonal damage marker). Seizures were noted in 33% of rickettsial infections, which was significantly higher than other bacterial groups. Markers of BBB leakage were increased in all patient groups. No differences were observed in albumin index and other examined biomarkers between *O*. *tsutsugamushi* and other rickettsial infections. There were no significant differences between scrub typhus and other rickettsial infections regarding demographics, outcome, CSF lactate, and protein or glucose levels. While *Orientia* and *Rickettsia spp*. account for 9% of all CNS infections in Laos, their acute phase neuroinflammation cannot be differentiated at the clinical level or with the neuroinflammation biomarkers tested [20].

### Rare acute neurologic manifestations of scrub typhus and SFR

Outside of ocular abnormalities and sensorineural hearing loss (discussed below), most rare acute neurologic reports can be divided into manifestations consistent with cerebral, cerebellar, or spinal injury. One striking case with both cerebral and cerebellar manifestations involved a 53-year-old female who was referred to a neurology clinic for progressive neurological deficits [21]. Prior to referral, she experienced 3 weeks of nonspecific symptoms with an eschar, the characteristic skin lesion evident on some scrub typhus patients and had been diagnosed with scrub typhus via polymerase chain reaction (PCR). This patient exhibited signs of cerebellar dysfunction (evidenced by positive Romberg, finger-nose-finger, and heel shin tests) and presented with lateral gaze palsy, drowsiness, and slurred speech. Computed tomography (CT) and MRI revealed intracranial hemorrhage, subarachnoid hemorrhage, and mass lesions in the right cerebellum, pons, and left midbrain. The patient progressed to a semicomatose state 3 hours after admission, and imaging studies revealed newly formed subarachnoid hemorrhage at the interpeduncular cistern and intraventricular hemorrhage at the left lateral ventricle. On hospital day 7, the patient became fully comatose and died. In this first report of hemorrhagic transformation of scrub typhus despite doxycycline treatment, the authors speculate that inflammation of vascular entities within the CNS may weaken the vascular walls, contributing to arterial rupture [21].

Cerebellar involvement in scrub typhus has been noticed in adult and pediatric patients [22,23]. One report discovered isolated cerebellar involvement in a 21-year-old man presenting with ataxia and slurred speech [24]. This patient had 5-day history of fever and rash, with 2-day history of inability to walk before presenting to the hospital. Diagnosis of scrub typhus was determined via Weil–Felix test and enzyme-linked immunosorbent assay (ELISA) targeting *O*. *tsutsugamushi*–specific IgM. CSF and CT findings were unremarkable, but MRI revealed isolated cerebellar cortical hyperintensity. Physicians noted a scrotal eschar and initiated empirical treatment with doxycycline, which rapidly improved the patient’s status. This patient survived and did not experience lasting sequelae. Cerebellar involvement in pediatric patients are reported mainly in “Letters to the Editor” format with limited clinical information. One report involved a 9-year-old boy presenting with fever, vomiting, headache, and left-sided swaying [23]. A physical exam revealed horizontal gaze nystagmus and truncal ataxia. MRI revealed focal cerebellar tonsil abnormalities and, since the child lived in an endemic area, scrub typhus was considered. Diagnosis of scrub typhus was made utilizing *O*. *tsutsugamushi*-specific IgM ELISA and PCR. Doxycycline treatment was initiated, and, within 48 hours, the patient defervesced; within one week, cerebellar symptoms resolved. Another report highlighted a 6-year-old girl presenting with 5-day history of fever and difficulty walking [25]. She experienced 3-day history of slurred speech and a single episode of tonic-clonic seizure, and her Weil–Felix test was positive, suggesting scrub typhus. Neurologic exam revealed scanning speech with positive cerebellar signs. Although her CSF analysis appeared unremarkable and no meningeal signs were present, imaging studies revealed isolated cerebellar folia effacement and early hydrocephalus.

Spinal cord involvement has been noted in one scrub typhus case. A 66-year-old man already receiving doxycycline treatment for scrub typhus (diagnosed with indirect fluorescent assay [IFA)] serum titers of 1:640) developed sudden urinary retention, bilateral ankle paresthesia, paraparesis, and loss of tendon reflexes [26]. CSF analyses revealed positive *O*. *tsutsugamushi*-specific IgG titers (1:110), and IFA revealed increased serum titers (1:2560). Spinal imaging revealed a high-intensity lesion and swelling around the T12 vertebrae, and acute transverse myelitis was diagnosed. The patient’s motor symptoms improved after steroid therapy, but urinary retention and the spinal lesion remained 6 months after discharge.

Ocular abnormalities have been observed in pediatric and adult scrub typhus patients. Initiation of steroid therapy leads to resolution of ocular findings across multiple case reports, implying an immune-mediated pathogenesis [27,28]. Optic neuritis, ocular flutter, and ophthalmoplegia have garnered interest [27–30]. One pediatric case involved an 8-year-old girl presenting with fever, headache, pain over the right eye, and rapidly progressing complete vision loss [28]. Physical examination findings were consistent with unilateral optic neuritis of the right eye and scrub typhus was diagnosed via IgM ELISA. Oral doxycycline and intravenous methylprednisone were initiated and continued for 5 days, allowing rapid symptom improvement. Follow-up 1-month posthospitalization revealed no residual visual defects. Ocular flutter was observed in a 9-year-old boy exhibiting cerebellar ataxia [27]. Physical examination revealed hepatosplenomegaly and a scrotal eschar. MRI of the brain was normal, and lymphocytic pleocytosis was observed in CSF studies. The IgM ELISA result was positive for scrub typhus, and intravenous doxycycline and dexamethasone were initiated. After 5 days of therapeutic intervention, the patient exhibited complete recovery. Ophthalmoplegia was noted in a 69-year-old farmer admitted to the hospital for scrub typhus [30]. This patient received doxycycline treatment for 5 days, at which point he was referred to neurology clinic due to complete ptosis and lack of directional movement of his left eye. Serum and CSF *O*. *tsutsugamushi* IgG was measured, with indirect IFA at 1:10,240 and 1:2,560, respectively. A nodular lesion in the anterior cavernous sinus and diffuse meningeal thickening was observed on MRI. After 10 days of continuing doxycycline treatment, the patient’s ocular symptoms resolved completely.

While tractable tinnitus and deafness may occur in approximately 20% of scrub typhus patients, no studies have examined the underlying mechanisms [31]. One case of sensorineural hearing loss due to SFR has recently been reported [32]. A pregnant 33-year-old woman presented to an outpatient clinic with 5-day history of fever, myalgia, headache, and maculopapular rash. She reported rapidly progressive hearing loss for 2 days, which was diagnosed as bilateral sensorineural deafness via audiometry. After a positive Weil–Felix test, she received azithromycin treatment for presumed rickettsial infection. Rickettsial infection was confirmed serologically 2 weeks later when *R*. *conorii* IgG titers were high (>1:450). Hearing impairment persisted for over 1 month, with a return to normal hearing 6 months after her illness.

### Sequelae of SFR neurological infection

One case report details sequelae of SFR in the timespan of years, in which a 9-year-old girl presented with fever and developed symptoms ranging from nausea and vomiting to polyarthritis and polymyositis within the span of 3 days [33]. About one week after initial presentation, she developed photophobia, dysphagia, and generalized seizures leading to obtundation. The patient exhibited severely impaired consciousness (Glasgow Coma Scale score of 8 or less) and multiple neurologic, dermatologic, and hepatologic abnormalities. However, lumbar puncture yielded findings within reference ranges. On day 10, IFA targeting anti–*R*. *rickettsii* IgM and amplification and sequencing targeting the 17-kDa fragment was performed and both were positive, indicating acute infection with *R*. *rickettsii*. She spent 5 days in critical care receiving intravenous chloramphenicol and was discharged 23 days later with anticonvulsant, neuroprotective, and physical therapies. Follow-up 10 years after discharge revealed lasting cerebral, cerebellar, and upper motor neuron signs, as well as symptoms that significantly impaired her quality of life, including sardonic facies, sialorrhea, ataxia, lower extremity muscular atrophy, and upper extremity spasticity.

While only a few reports detail neurologic sequelae of SFR in the timespan of months, all cases reveal some form of lasting neurological deficits. In one case, delayed doxycycline treatment of a 7-year-old girl with Rocky Mountain spotted fever in the United States led to hospitalization [34]. Upon admission, empiric ceftriaxone, vancomycin, and doxycycline were initiated; however, rapidly declining respiratory capacity necessitated intubation and mechanical ventilation. On hospital day 5, imaging studies revealed hyperintense lesions in the subcortical, periventricular, deep white matter, and corpus callosum, consistent with cytotoxic edema resulting from ongoing tissue injury. On hospital day 16, the patient was extubated and exhibited dystonic neck posturing, left arm flexion, and generalized loss of tone. Imaging studies then revealed an increase in number and size of lesions, with a classical “starry sky” appearance and new lesions in the basal ganglia. Together, these imaging findings indicated ongoing cerebral damage despite appropriate treatment. Steroid treatment was initiated and did not yield symptom improvement. On day 24 of illness, a diagnosis of *R*. *rickettsii* infection was made based upon IgM (1:256) and IgG (1:1024) titers. About 2 months after admission, residual left arm spasticity and significant language and communication deficits remained. The ongoing cerebral injury in the face of antibiotic treatment led the authors to recommend coadministration of antiinflammatory agents and antibiotics at disease outset. The authors speculated that the initial delay in treatment led to a robust antibody response, which may have caused small-vessel vasculitis and cerebral injury.

Another report recommended the coadministration of antiinflammatory agents along with antibiotics during SFR infection [35]. A case of Mediterranean spotted fever (CSF IgG 1:320, IgM 1:10 to *R*. *conorii*) in a 63-year-old woman led to chronic inflammatory demyelinating polyneuropathy (CIDP). This patient exhibited relapses over the course of months, all of which responded to either intravenous immunoglobulin or steroid therapies. Four months after the initial relapse of CIDP, the patient exhibited difficulties in fine hand movements as well as areflexia. This led the authors to recommend immunologic treatment in addition to antibiotic therapy in initial treatment of *R*. *conorii*.

In an American case report, a 27-month-old boy developed a centripetally spreading petechial rash [36]. A tick was removed, and he was treated with amoxicillin and, then, ceftriaxone over the course of about 4 days. About 8 days after onset of rash, he developed reduced consciousness and experienced a generalized clonic seizure. After intubation, neurologic exam revealed intact brainstem reflexes, increased peripheral reflexes, positive bilateral Babinski sign, and rigid extensor posturing to noxious stimuli. Electroencephalogram studies revealed discreet seizures originating from the right temporal and left parieto-occipital lobes, indicating potential subclinical status epilepticus. Lorazepam and levetiracetam administration aborted the seizures. Imaging studies revealed multifocal punctate lesions, consistent with a starry sky pattern. Lumbar puncture revealed elevated protein. Serum IFA revealed positive *Rickettsia* IgM (1:1,024) and IgG (1:64) titers, at which point treatment was narrowed to doxycycline. After appropriate treatment, laboratory studies returned to baseline after approximately one week. Two months after discharge, the boy experienced some lingering neurological deficits necessitating a gastric tube and was unable to speak.

### Limitations in clinical studies

These clinical studies have several inherent limitations; they are either case reports or retrospective studies with relatively small sample sizes, introducing a higher potential for sampling errors. In retrospective studies, scrub typhus patients were often identified utilizing paired serum samples and thorough clinical notes [18]. However, complete clinical records and appropriate samples were not always available, which means many cases may not be diagnosed or reported. Clinical studies identify the result of severe infection and generally cannot define the tipping point from mild to severe disease. It is also unclear whether neurological manifestations observed in patients are direct results of *O*. *tsutsugamushi* or rickettsial neuroinvasion, an immunologically mediated process, or ischemic damage arising during infection. Therefore, animal model–based research is in urgent need to better understand scrub typhus and SFR pathogenesis and neuroinflammation.

## Molecular pathogenesis in experimental animal models

### *O*. *tsutsugamushi* infection in murine models

Scrub typhus severity is dependent on host immune status and the *O*. *tsutsugamushi* strains involved; the Karp serotype (OtK) is the most prevalent strain in patients, accounting for approximately 40% of infections in endemic countries [2]. OtK is also the most virulent serotype in experimental animals, causing lethal or sublethal infections in outbred and inbred mice, depending on the inoculation doses and routes. In 1948, a study for OtK persistence in outbred albino Swiss mice suggested that bacteria in brain homogenates remained infective for more than 610 days postinfection; however, dedicated study for the mechanisms for bacterial persistence was lacking [37]. In 2014, Keller and colleagues used inbred BALB/c mice and provided evidence for dissemination of OtK from footpad inoculation site to draining lymph nodes and visceral organs (lungs, brain, etc.) [38]. Using this self-limiting model of scrub typhus, these authors documented astrocyte activation in the CNS and increased iNOS expression and infiltration of T-cells and infected macrophages into the cerebral parenchyma, implying a breakdown of the BBB during disease progression. Recently, our group developed an intradermal (i.d.) inoculation model in C57BL/6 mouse ears for kinetic studies of bacterial dissemination and cellular and antibody responses during acute versus persistent infection [39]. While skin-inoculation models are useful for studying bacterial dissemination and self-limiting scrub typhus or for vaccine-based studies, they lack certain pathological features in human cases or CNS alterations during severe infection [39].

To address these shortcomings, we developed a severe scrub typhus model for endothelium-targeted OtK infection in C57BL/6 mice, which permits mechanistic examination of pathogenesis during lethal versus sublethal infections and the magnitude of CNS alterations [40,41]. We showed significant gene up-regulation for immune recognition and inflammatory responses throughout the course of infection in brain tissues. In particular, transcription of genes related to pathogen-pattern recognition (TLR2, TLR4, and TLR9), type-1 responses (IFNγ, TNFα, CXCL9, and CXCR3), and endothelial stress and/or damage (indicated by increased angiopoietins [Ang2 and Ang2/1 ratio] but a rapid down-regulation of Tie2 [an endothelial tyrosine kinase receptor]) were significantly up-regulated during a lethal infection. Sublethal infection displayed similar trends, implying the development of type 1–skewed proinflammatory responses in infected brains, independent of time and disease outcomes [38]. Meningitis and punctate hemorrhagic lesions were observed in both groups, and histological changes were more common in lethal outcomes. At 6 to 10 days of lethal infection, the cortex and cerebellum displayed increased ICAM-1–positive staining in vascular cells. This resulted in increased detection of CD45^+^ leukocytes, CD3^+^ T cells, and IBA1^+^ phagocytes (microglia and infiltrating macrophages in the CNS) within the parenchyma and activation of GFAP^+^ astrocytes but a significant loss of tight junction staining (occludin), implying progressive activation and damage of the endothelium. Bacteria were infrequently observed in the brain but were detectable within phagocytic cells.

To define neuroinflammation associated with scrub typhus, we used NanoString technology to quantify the transcripts of host immune genes in brain tissues of lethally infected C57BL/6 mice at zero versus 10 days, as described previously [41,42]. This nonamplification-based technology reduces any artificial biases introduced through PCR amplification, allowing simultaneous detection of more than 500 immunology-associated genes. **Table 1** highlights the significantly up-regulated genes at day 10; some differentially expressed genes were validated from previous qRT-PCR reports [38,40]. Of note, there are 367-fold to 2663-fold increases in CXCR3 ligands (Cxcl9, Cxcl0, and Cxcl11), 48-fold to 273-fold increases in inflammatory chemokines (Ccl3, Ccl4, and Ccl8), and marked increases in type 1-cytokines such as IFNγ (68-fold) and TNFα (111-fold). In addition to iNOS up-regulation known to be relevant to OtK and *Rickettsia* neuropathogenesis [38,43,44], we also detected a marked up-regulation of cytotoxicity- and killing-related genes (granzyme A and B and members of the TNF superfamily), implying cellular and/or tissue damage during severe infection with OtK. Involvement of CD8^+^ T cells in scrub typhus-associated neuroinflammation is highly possible based on the detection of CD8α/β chains (**Table 1**); the cellular sources of granzyme A and B and the functional roles of CD8^+^ T-cells in inflamed brains warrant further investigation. The findings of endothelial activation (L-selectin, ICAM, PECAM, and integrin up-regulation) were consistent with our previous findings with immunostaining of the brain tissues and damage of brain microvasculature during OtK infection [41]. These findings may help explain leukocyte recruitment and BBB impairment in severe scrub typhus patients. We also found complement involvement (especially factors B and C3) in infected brain tissues; however, complement-mediated neuropathogenesis has not been explored in scrub typhus, although some studies have been done for rickettsial infections [44]. A single publication was found describing a lethal footpad infection of DRAGA humanized C3H/HeJ mice with OtK [45]. This model showed very similar pathologies and disease when compared with human cases with significant bacterial replication in the liver, as well as the lung, leading to acute respiratory distress; however, the authors did not observe any pathologic changes in the brain.

**Table 1:** Mouse Brain Tissue Gene Expression (day 10 versus mock).

Chemokine and Cytokine Gene Expression				Cytotoxicity and Killing-Related Gene Expression			
Gene	Alias / Encoded Proteins	Accession #	Fold Change	GGenene	Alias / Encoded Proteins	Accession #	Fold Change
**Cxcl10**	CXCL10/IFN-γ-inducible protein 10	NM_021274.1	**2663.13**^a^	**Gzma**	Granzyme A	NM_010370.2	**612.26**^b^
**Cxcl9**	CXCL9/IFN-γ-inducible protein 9	NM_008599.2	**449.64** ^a^	**Gzmb**	Granzyme B	NM_013542.2	**459.26** ^b^
**Cxcl11**	CXCL11/IFN-γ-inducible protein 11	NM_019494.1	**367.24**^b^	**Cybb**	Cytochrome b245, β polypeptide	NM_007807.2	**186.15** ^a^
**Ccl8**	CCL8/MCP-2	NM_021443.2	**273.87** ^b^	**Cd8a**	T-cell surface glycoprotein 8, α chain	NM_001081110.2	**172.08** ^b^
**Il1rn**	IL-1 receptor antagonist protein 1	NM_031167.5	**195.78** ^b^	**Tnf**	Tumor Necrosis Factor-α	NM_013693.1	**111.42** ^a^
**Il18rap**	IL-18 receptor accessory protein precursor	NM_010553.2	**173.43** ^b^	**Cd244**	NK Cell Receptor 2B4	NM_018729.2	**93.05** ^b^
**Ccl4**	CCL4/MIP-1β (Macrophage Inflammatory Protein 1β)	NM_013652.1	**121.37** ^a^	**Cd27**	TNF receptor superfamily member 7	NM_001042564.1	**45.53** ^a^
**Cxcr6**	CXCR6	NM_030712.4	**86.60** ^b^	**Cd8b1**	T-cell surface glycoprotein 8, β chain	NM_009858.2	**43.79** ^b^
**Il18r1**	IL-18 receptor 1 precursor	NM_001161842.1	**86.27** ^a^	**Pdcd1**	Programmed Cell Death protein 1	NM_008798.1	**35.70** ^a^
**Il2rg**	IL-2 receptor γ chain	NM_013563.3	**69.85** ^a^	**Cd247**	T-cell surface glycoprotein, ζ chain	NM_001113391.2	**35.20** ^b^
**Ifng**	Interferon γ	NM_008337.1	**68.51** ^b^	**Fasl**	Fas Ligand	NM_010177.3	**34.51** ^a^
**Il21r**	IL-21 receptor	NM_021887.1	**59.03** ^b^	**Cd40lg**	CD40 Ligand	NM_011616.2	**21.33** ^a^
**Irf7**	Interferon regulatory factor 7	NM_016850.2	**53.31** ^b^	**B2m**	Beta-2 microglobulin	NM_009735.3	**14.39** ^b^
**Ccl3**	CCL3/MIP-1α (Macrophage Inflammatory Protein 1α)	NM_011337.1	**48.91** ^b^	**Nos2**	Nitrous Oxide Synthase, inducible	NM_010927.3	**11.52** ^a^
**Il12rb1**	IL-12 receptor β1 subunit	NM_008353.2	**41.57** ^a^	**Pdcd1lg2**	Programmed Cell Death protein 1 Ligand 2	NM_021396.2	**9.57** ^a^
**Il12rb2**	IL-12 receptor β2 subunit	NM_008354.3	**40.56** ^a^	**Tnfrsf1b**	TNF receptor superfamily member 1b	NM_011610.3	**8.36** ^a^
**Irf1**	Interferon Regulatory Factor 1	NM_008390.1	**27.14** ^b^	**Ltb**	Lymphotoxin β	NM_008518.2	**4.49** ^a^
**Cxcl1**	CXCL1	NM_008176.1	**26.00** ^a^	**Tnfrsf13b**	TNF receptor superfamily member 13b (TACI)	NM_021349.1	**3.19** ^a^
**Il2ra**	IL-2 receptor subunit α	NM_008367.2	**25.94** ^a^	**Cell Adhesion Molecule Gene Expression**			
**Il27ra**	IL-27 receptor subunit α	NM_016671.3	**12.85** ^a^	**Itgal**	Integrin subunit- α L	NM_008400.2	**604.17** ^b^
**Ifi35**	Interferon-induced 35kDa protein	NM_027320.4	**11.98** ^b^	**Sell**	L-selectin	NM_001164059.1	**244.61** ^a^
**Irf8**	Interferon Regulatory Factor 8	NM_008320.3	**11.48** ^b^	**Cd74**	Invariant Polypeptide of MHC Class II	NM_001042605.1	**199.41** ^a^
**Il6**	IL-6	NM_031168.1	**11.34** ^a^	**Cd226**	PTA1 (Platelet & T cell activation antigen 1)	NM_001039149.1	**115.72** ^b^
**Ccl19**	CCL19/MIP-3β (Macrophage Inflammatory protein 3β)	NM_011888.2	**11.20** ^a^	**Irgm1**	Immunity-related GTPase family M protein 1	NM_008326.1	**50.48** ^a^
**Il10**	IL-10	NM_010548.1	**10.58** ^b^	**Icam1**	Intercellular Adhesion Molecule 1	NM_010493.2	**24.15** ^b^
**Ifit2**	Interferon-Induced Protein with Tetratricopeptide Repeats 2	NM_008332.2	**9.14** ^b^	**Pecam1**	Platelet/Endothelial Cell Adhesion Molecule 1	NM_008816.2	**3.26** ^a^
**Irf5**	Interferon Regulatory Factor 5	NM_012057.3	**8.99** ^a^	**Cdh5**	Vascular Endothelial Cadherin	NM_009868.3	**2.97** ^a^
**Ccrl1**	CCRL1/ACKR4 (Atypical Chemokine Receptor 4)	NM_145700.2	**6.29** ^b^	**Ceacam1**	Carcinoembryonic Antigen-Related Cell Adhesion Molecule 1	NM_001039185.1	**2.11** ^a^
**Ccrl2**	CCRL2/ACKR5 (Atypical Chemokine Receptor 5)	NM_017466.4	**6.06** ^b^	**Complement Gene Expression**			
**Il9**	IL-9	NM_008373.1	**4.87** ^a^	**Cfb**	Factor B	NM_008198.2	**1151.21** ^a^
**Il10ra**	IL-10 receptor α chain	NM_008348.2	**4.74** ^a^	**C3**		NM_009778.2	**606.81** ^a^
**Il6ra**	IL-6 receptor α chain	NM_010559.2	**4.33** ^a^	**C1s**		NM_144938.2	**18.19** ^a^
**Il16**	IL-16	NM_010551.3	**3.69** ^a^	**C1qa**		NM_007572.2	**6.64** ^a^
**Il4ra**	IL-4 receptor α chain	NM_001008700.3	**2.88** ^a^	**C1qb**		NM_009777.2	**6.29** ^a^
**Il15**	IL-15	NM_008357.2	**1.97** ^a^	**Cfp**	Properdin	NM_008823.3	**2.59** ^b^
**Il10rb**	IL-10 receptor β chain	NM_008349.5	**1.55** ^a^	**Cfh**	Factor H	NM_009888.3	**2.11** ^b^

All listed cytokines/chemokines are proinflammatory with the exception of few notable regulatory/type 2 cytokines: Il21r, Il2ra, Il10, Il10ra, Il4ra, and Il10rb.

^a^ denotes *p* < 0.05

^b^ denotes *p* < 0.01.

Overall, these CNS alterations are consistent with type 1-skewed, type 2-suppressed responses in lethally infected mouse lungs [40–42]. Our findings collectively support a notion of vascular activation and dysfunction during OtK infection and provide new clues for further examination of tissue-specific immune mechanisms. The trends of mouse immune responses from OtK infection models resemble those in rickettsial infections in mice (see below), implying shared mechanisms in CNS involvement [46]. Furthermore, pathological examination of tissues highlighted a marked increase in population of the resident microglial cells over that of infiltrating macrophages [47]. While recent molecular and immunological studies have investigated the involvement of phagocytic cells in the brain, a distinction has not been made between resident microglial cells and infiltrating monocytes [38,40,41].

### *Rickettsia* Infection in Murine Models

The mechanisms of neurological disease during human infection remain unknown [48]. Since rickettsia are obligate intracellular bacteria, hypotheses include direct or indirect neuroinvasion or immunologically mediated neuronal damage [48]. Rickettsial diseases have been modeled with the C3H/HeN mouse strain that effectively recapitulates classical SFR disease [49]. Resistance to *R*. *conorii* has also been shown for RAG1^-/-^ C57BL/6 mice (deficient B and T cell functions) for up to 30 days, highlighting the knowledge gap concerning the role of adaptive immunity in this model [50]. However, the persistence of typhus group rickettsiae has recently been reported in RAG1^**-/-**^ C57BL/6 model [43]. Subcutaneous or intravenous inoculation with *R*. *typhi* in wild-type or RAG1^-/-^ mice generated no significant acute disease; however, neuronal recrudescence was observed in RAG1^-/-^ mice at 3–4 months postinfection, leading to sudden paralysis and death, whereas immunocompetent mice are persistently infected for at least a year, with no recrudescence [43]. These neurological signs were associated with microglial expansion, neuronal cell death, and infiltration of *Rickettsia*-containing, iNOS-expressing macrophages in C57BL/6 mice. However, the mechanisms for adaptive immune suppression of recrudescence has not been studied. Supporting the role of adaptive immunity in preventing persistence and recrudescence is the evidence that adoptive transfer of *Rickettsia*-specific CD4^+^ or CD8^+^ cells to RAG1^-/-^ mice protects from lethal challenge and effectively clears *R*. *typhi* from the CNS if administered early, although the mechanisms for CD4^+^ and CD8^+^ cells are distinct [44]. CD4^+^ cell transfer was determined to induce IBA^+^ cells to express iNOS, eliminating the intracellular bacteria, similar to the study with the footpad inoculation with *O*. *tsutsugamushi* [38,44].

It is known that IL-1β and TNFα augment permeability of infected brain endothelial cells following in vitro infection with *R*. *rickettsii* by interfering with the p120/β-catenin adherens junctions between cells, and that these cytokines are actively expressed during OtK infection in vivo [41,50]. Loss of tight junction integrity was independent from NO generated by the cell during infection [50]. Even though p38 and NF-κB activation is evident in this in vitro model, several of the downstream chemokines (Macrophage Inflammatory Protein [MIP]-1α/CCL3, MCP-1/CCL2, and IL-8) do not show any up-regulation in SV-HCECs (immortalized human cerebral microvascular endothelial cells), but infection of HUVECs (human umbilical veil endothelial cells) with *R*. *rickettsii* or *O*. *tsutsugamushi* does induce their expression [51–53]. This may be a limitation of the SV40-transformed cell line, or an immunological difference between *R*. *rickettsii* and *O*. *tsutsugamushi*. However, these chemokines (MIP-1α/CCL3, MCP-1/CCL2, IL-8) have not yet been assayed in the brain tissues. In vitro infection of rat neurons shows that *Rickettsia spp*. significantly reduces the intracellular levels of ATP and induces cellular apoptosis in those neurons and does so at a greater degree than when infecting endothelial cells; however, the underlying mechanisms were not elucidated [54,55].

### Limitations of murine models

There are several different mouse strains and models for rickettsial diseases, including C3H/HeN, BALB/c, and C57BL/6 [8,40,49,56]. The most common models are C3H/HeN mice for *Rickettsia* and C57BL/6 mice for scrub typhus. One key factor influencing the model’s effectiveness in replicating the natural course of infection is the route of inoculation. Early models of scrub typhus frequently used intraperitoneal inoculation. This route causes a fulminant peritonitis with significant hepatic and splenic infection with little to no pulmonary involvement and infection of peritoneal macrophages: manifestations of scrub typhus not resembling the natural disease [57]. While intradermal and subcutaneous inoculation routes do resemble the natural mode of infection, and they are useful for investigating early immune responses to the bacteria, they fail to produce lethal infections [38,39,58]. For both pathogens, intravenous inoculation through the tail can mimic the hematogenous dissemination and systemic involvement of all major organs [40]. For OtK, titration of the dose to determine a consistent LD_50_ is difficult and infection will usually yield a sublethal or an 80% to 100% lethal outcome when the concentration is between 1-5x10^4^ FFU, in our hands. **Table 2** summarizes the molecular pathogenesis and animal models of these diseases. While murine models may present a partial picture of human disease, the characteristic cytokines observed in human scrub typhus (increased TNFα and IL-10) are also observed in an IV inoculation of mice, consistent with progression to interstitial pneumonia and acute respiratory distress [59,60]. The correlation between murine and human neuropathology, however, remains elusive due to a dearth of studies performing histopathologic investigation of fatal patient cases. Assessing how closely murine models parallel the neurological manifestations of scrub typhus observed in patients (coma, ataxia, delirium, etc.) presents major challenges.

**Table 2:** Summary of molecular pathogenesis in mouse models.

Affected Cell Types in the Brain		SFG *Rickettsia*	*Orientia tsutsugamushi*	References
	Endothelial cells	Support replication	Support replication	[49, 61]; [62]
	Microglia	Support replication (*R*. *typhi*)	Activation	[44]; [38, 41]
	Neurons	Cell death observed	No data	[54]
	Astrocytes	Activation	Activation	[20]; [38, 41]
	Infiltrating Macrophages	Support replication	Support replication	[44]; [38, 60]
				
**Key Cytokines**				
	TNFα	Up-regulated	Strongly up-regulated	[63]; This manuscript
	IFN-γ	Up-regulated	Strongly up-regulated	[63]; This manuscript
	IL-6	Up-regulated	Up-regulated	[63]; This manuscript
	IL-1β	Up-regulated	Up-regulated	[64]
	MIP-1α	Up-regulated	Strongly up-regulated	[63] This manuscript

## Closing remarks

Neurological manifestations of disease are severe and potentially debilitating to patients during acute infection and chronic illness. Improving clinical awareness and appropriate empiric treatment in endemic areas presents a challenge but would potentially reduce the neurologic complications of both diseases. Fundamental questions regarding critical inflammatory cell populations have arisen from clinical studies and animal models studying neuroinflammation in scrub typhus and SFR (see Key Learning Points). Much effort is currently placed on uncovering answers to driving molecular mechanisms of neuroinflammation during both diseases (see Top 5 Papers). A combined use of unbiased, targeted quantification platforms such as NanoString technology and immunological approaches including immunofluorescent staining at the tissue and/or single cell level would allow detection of a panel of host gene and protein targets. A greater understanding of disease neuropathogenesis could lead to new therapeutic targets or strategies and significantly improve outcomes for patients presenting with neurological scrub typhus or SFR.

**Key Learning Points**

- Scrub typhus and SFR can display neurological manifestations, with CSF studies in both diseases yielding findings of aseptic meningitis or tubercular meningitis.
- Scrub typhus and SFR cannot be differentiated based on current clinically used neuroinflammatory markers: albumin index, GFAP, s100b, neuron-specific enolase, or total tau protein.
- Coadministration of steroid therapy with antibiotics during early infection has been found to be beneficial for reducing neurological signs and symptoms in both scrub typhus and SFR.
- Murine models for these diseases can be refractory or very susceptible, depending on the routes of inoculation and mouse strains. Intravenous inoculation recapitulates hallmarks of natural infection (i.e., hematogenous dissemination and systemic involvement).
- Scrub typhus and SFG Rickettsia have similar cytokine and cellular response profiles, highlighted by increases in TNFα, IFNγ, and other type 1-skewed proinflammatory responses.

**Top Five Papers**

1. Dittrich S, Sunyakumthorn P, Rattanavong S, Phetsouvanh R, Panyanivong P, Sengduangphachanh A, Phouminh P, Anantatat T, Chanthongthip A, Lee SJ, Dubot-Pérès A. Blood–brain barrier function and biomarkers of central nervous system injury in Rickettsial versus other neurological infections in Laos. The American journal of tropical medicine and hygiene. 2015 Aug 5;93(2):232–7.
2. Dittrich S, Rattanavong S, Lee SJ, Panyanivong P, Craig SB, Tulsiani SM, Blacksell SD, Dance DA, Dubot-Pérès A, Sengduangphachanh A, Phoumin P. Orientia, rickettsia, and leptospira pathogens as causes of CNS infections in Laos: a prospective study. The Lancet Global Health. 2015 Feb 1;3(2):e104-12.
3. Soong L, Shelite TR, Xing Y, Kodakandla H, Liang Y, Trent BJ, et al. Type 1-skewed neuroinflammation and vascular damage associated with Orientia tsutsugamushi infection in mice. PLoS Negl Trop Dis. 2017;11(7):e0005765-e.
4. Rydkina E, Turpin LC, Sahni SK. Rickettsia rickettsii infection of human macrovascular and microvascular endothelial cells reveals activation of both common and cell type-specific host response mechanisms. Infect Immun. 2010;78(6):2599–606.
5. Dzul-Rosado KR, Lugo-Caballero C, Salcedo-Parra A, López-Soto RD, Faccini-Martínez ÁA. Long term neurologic sequelae in a Mexican rocky mountain spotted fever case. The Brazilian Journal of Infectious Diseases. 2019 Mar 1;23(2):121–3.

## References

[1] Xu G, Walker DH, Jupiter D, Melby PC, Arcari CM. A review of the global epidemiology of scrub typhus. PLoS Negl Trop Dis. 2017;11(11):e0006062 10.1371/journal.pntd.0006062 29099844 PMC5687757

[2] Kelly DJ, Fuerst PA, Ching W-M, Richards AL. Scrub typhus: The geographic distribution of phenotypic and genotypic variants of *Orientia tsutsugamushi*. Clinical Infectious Diseases. 2009;48(Supplement_3):S203–S30. 19220144 10.1086/596576

[3] Kim G, Ha N-Y, Min C-K, Kim H-I, Yen NTH, Lee K-H, et al Diversification of *Orientia tsutsugamushi* genotypes by intragenic recombination and their potential expansion in endemic areas. PLoS Negl Trop Dis. 2017;11(3):e0005408 10.1371/journal.pntd.0005408 28248956 PMC5348041

[4] Weitzel T, Martínez-Valdebenito C, Acosta-Jamett G, Jiang J, Richards AL, Abarca K. Scrub Typhus in Continental Chile, 2016–2018. Emerg Infect Dis. 2019;25(6):1214–7. 10.3201/eid2506.181860 30835200 PMC6537721

[5] Taylor AJ, Paris DH, Newton PN. A Systematic Review of Mortality from Untreated Scrub Typhus (*Orientia tsutsugamushi*). PLoS Negl Trop Dis. 2015;9(8):e0003971 10.1371/journal.pntd.0003971 26274584 PMC4537241

[6] Bonell A, Lubell Y, Newton PN, Crump JA, Paris DH. Estimating the burden of scrub typhus: A systematic review. PLoS Negl Trop Dis. 2017;11(9):e0005838–e. 10.1371/journal.pntd.0005838 28945755 PMC5634655

[7] Abdad MY, Abou Abdallah R, Fournier PE, Stenos J, Vasoo S. A Concise Review of the Epidemiology and Diagnostics of Rickettsioses: Rickettsia and Orientia spp. J Clin Microbiol. 2018;56(8). 10.1128/JCM.01728-17 PMC6062794 29769278

[8] Walker DH, Dumler JS. The role of CD8 T lymphocytes in rickettsial infections. Semin Immunopathol. 2015;37(3):289–99. 10.1007/s00281-015-0480-x 25823954 PMC4458380

[9] Faccini-Martínez Á, García-Álvarez L, Hidalgo M, Oteo JA. Syndromic classification of rickettsioses: an approach for clinical practice. Int J Infect Dis. 2014;28:126–39. 10.1016/j.ijid.2014.05.025 25242696

[10] Dubot-Peres A, Mayxay M, Phetsouvanh R, Lee SJ, Rattanavong S, Vongsouvath M, et al Management of central nervous system infections, Vientiane, Laos, 2003–2011. Emerg Infect Dis. 2019;25(5):898–910. 10.3201/eid2505.180914 31002063 PMC6478220

[11] Sadanandane C, Jambulingam P, Paily KP, Kumar NP, Elango A, Mary KA, et al Occurrence of *Orientia tsutsugamushi*, the etiological agent of scrub typhus in animal hosts and mite vectors in areas reporting human cases of acute encephalitis syndrome in the Gorakhpur region of Uttar Pradesh, India. Vector Borne Zoonotic Dis. 2018;18(10):539–47. 10.1089/vbz.2017.2246 30016222

[12] Lee HS, Sunwoo JS, Ahn SJ, Moon J, Lim JA, Jun JS, et al Central Nervous System Infection Associated with. Am J Trop Med Hyg. 2017;97(4):1094–8. 10.4269/ajtmh.17-0077 28820719 PMC5637598

[13] Jain P, Prakash S, Tripathi PK, Chauhan A, Gupta S, Sharma U, et al Emergence of *Orientia tsutsugamushi* as an important cause of Acute Encephalitis Syndrome in India. PLoS Negl Trop Dis. 2018;12(3):e0006346 10.1371/journal.pntd.0006346 29590177 PMC5891077

[14] Dittrich S, Rattanavong S, Lee SJ, Panyanivong P, Craig SB, Tulsiani SM, et al Orientia, rickettsia, and leptospira pathogens as causes of CNS infections in Laos: a prospective study. Lancet Glob Health. 2015;3(2):e104–12. 10.1016/S2214-109X(14)70289-X 25617190 PMC4547322

[15] WHO. WHO–recommended standards for surveillance of selected vaccine-preventable diseases. 2003.

[16] Misra UK, Kalita J, Mani VE. Neurological manifestations of scrub typhus. J Neurol Neurosurg Psychiatry. 2015;86(7):761–6. 10.1136/jnnp-2014-308722 25209416

[17] Maitra S, Chakravarty UK, Ray K. Scrub Typhus Meningismus: A Diagnostic Dilemma. Journal of Clinical and Diagnostic Research 2020 10.7860/JCDR/2020/19159.13882 32662777 PMC7357717

[18] Lee HS, Sunwoo JS, Ahn SJ, Moon J, Lim JA, Jun JS, et al Central nervous system infection associated with *Orientia tsutsugamushi* in South Korea. Am J Trop Med Hyg. 2017;97(4):1094–8. 10.4269/ajtmh.17-0077 28820719 PMC5637598

[19] Naoi T, Shimazaki H, Sawada M. The Rapid Effectiveness of Minocycline against Scrub Typhus Meningoencephalitis. Intern Med. 2016;55(7):805–9. 10.2169/internalmedicine.55.5304 27041169

[20] Dittrich S, Sunyakumthorn P, Rattanavong S, Phetsouvanh R, Panyanivong P, Sengduangphachanh A, et al Blood-Brain Barrier Function and Biomarkers of Central Nervous System Injury in Rickettsial Versus Other Neurological Infections in Laos. Am J Trop Med Hyg. 2015;93(2):232–7. 10.4269/ajtmh.15-0119 26055741 PMC4530739

[21] Kim HC, Yoon KW, Yoo DS, Cho CS. Hemorrhagic Transformation of Scrub Typhus Encephalitis: A Rare Entity. Clin Neuroradiol. 2015;25(4):415–8. 10.1007/s00062-014-0348-9 25373351

[22] Kaiser RS, Khemka A, Roy O, Das S, Datta K. An Unusual Etiology of Cerebellar Ataxia. Child Neurol Open. 2020;7:2329048X20907754. 10.1177/2329048X20907754 PMC7081463 32215279

[23] Didel S, Basha MA, Biswal M, Suthar R, Sankhyan N. Acute Cerebellitis in a Child With Scrub Typhus. Pediatr Infect Dis J. 2017;36(7):696–7. 10.1097/INF.0000000000001524 28604564

[24] Bhoil R, Kumar S, Sood RG, Bhoil S, Verma R, Thakur R. Cerebellitis as an atypical manifestation of scrub typhus. Neurology. 2016;86(22):2113–4. 10.1212/WNL.0000000000002717 27242380

[25] Bhat MD, Vykuntaraju KN, Acharya UV, Ramaswamy P, Prasad C. Isolated Cerebellitis in Scrub Typhus. Indian J Pediatr. 2015;82(11):1067–8. 10.1007/s12098-015-1784-5 25990597

[26] Ryu HS, Moon BJ, Park JY, Kim SD, Seo SK, Lee JK. Acute transverse myelitis following scrub typhus: A case report and review of the literature. J Spinal Cord Med. 2018:1–4. 10.1080/10790268.2017.1420538 PMC7480440 29350608

[27] Kasinathan A, Suthar R, Sahu JK, Sankhyan N, Nallasamy K. Ocular Flutter in Scrub Typhus. J Pediatr. 2019;204:315 10.1016/j.jpeds.2018.08.025 30266504

[28] Jessani LG, Gopalakrishnan R, Kumaran M, Devaraj V, Vishwanathan L. Scrub Typhus Causing Unilateral Optic Neuritis. Indian J Pediatr. 2016;83(11):1359–60. 10.1007/s12098-016-2169-0 27271884

[29] Bae DW, An JY. Anti-aquaporin-4 Antibody Positive Optic Neuritis Following Scrub Typhus in an Elderly Female. Neurologist. 2018;23(6):183–4. 10.1097/NRL.0000000000000196 30379739

[30] Kim J, Kim JS, Shin DI, Lee SH, Lee SS, Choi SY. Ophthalmoplegia Due to Scrub Typhus. J Neuroophthalmol. 2015;35(3):284–6. 10.1097/WNO.0000000000000259 25993123

[31] Premaratna R, Chandrasena TG, Dassayake AS, Loftis AD, Dasch GA, de Silva HJ. Acute hearing loss due to scrub typhus: a forgotten complication of a reemerging disease. Clin Infect Dis. 2006;42(1):e6–8. 10.1086/498747 16323083

[32] Kariyawasam AGTA, Palangasinghe DR, Fonseka CL, De Silva PUT, Kanakkahewa TE, Dahanayaka NJ. Bilateral Sensorineural Deafness in a Young Pregnant Female Presenting with a Fever: A Rare Complication of a Reemerging Disease-Spotted Fever Group Rickettsioses. Case Rep Infect Dis. 2019;2019:5923146 10.1155/2019/5923146 31019815 PMC6452535

[33] Dzul-Rosado KR, Lugo-Caballero C, Salcedo-Parra A, López-Soto RD, Faccini-Martínez Á. Long term neurologic sequelae in a Mexican rocky mountain spotted fever case. Braz J Infect Dis. 2019;23(2):121–3. 10.1016/j.bjid.2019.04.006 31103437 PMC9425664

[34] Sun LR, Huisman TA, Yeshokumar AK, Johnston MV. Ongoing Cerebral Vasculitis During Treatment of Rocky Mountain Spotted Fever. Pediatr Neurol. 2015;53(5):434–8. 10.1016/j.pediatrneurol.2015.07.003 26294045

[35] Tzanetakos D, Papadopoulou M, Kanellopoulos D, Mamali M, Safarikas M, Katsianos D, et al Chronic inflammatory demyelinating polyneuropathy associated with *Rickettsia conorii*: First case report. J Neurol Sci. 2016;371:60–1. 10.1016/j.jns.2016.10.015 27871450

[36] Bradshaw MJ, Lalor KB, Vu N, Pruthi S, Bloch KC. Child Neurology: Rocky Mountain spotted fever encephalitis. Neurology. 2017;88(11):e92–e5. 10.1212/WNL.0000000000003722 28289173

[37] Fox JP. The long persistence of *Rickettsia orientalis* in the blood and tissues of infected animals. The Journal of Immunology. 1948;59(2):109–14. 18864084

[38] Keller CA, Hauptmann M, Kolbaum J, Gharaibeh M, Neumann M, Glatzel M, et al Dissemination of *Orientia tsutsugamushi* and inflammatory responses in a murine model of scrub typhus. PLoS Negl Trop Dis. 2014;8(8):e3064–e. 10.1371/journal.pntd.0003064 25122501 PMC4133189

[39] Soong L, Mendell NL, Olano JP, Rockx-Brouwer D, Xu G, Goez-Rivillas Y, et al An intradermal inoculation mouse model for immunological investigations of acute scrub typhus and persistent infection. PLoS Negl Trop Dis. 2016;10(8):e0004884–e. 10.1371/journal.pntd.0004884 27479584 PMC4968841

[40] Shelite TR, Saito TB, Mendell NL, Gong B, Xu G, Soong L, et al Hematogenously disseminated *Orientia tsutsugamushi*-infected murine model of scrub typhus [corrected]. PLoS Negl Trop Dis. 2014;8(7):e2966–e. 10.1371/journal.pntd.0002966 25010338 PMC4091938

[41] Soong L, Shelite TR, Xing Y, Kodakandla H, Liang Y, Trent BJ, et al Type 1-skewed neuroinflammation and vascular damage associated with *Orientia tsutsugamushi* infection in mice. PLoS Negl Trop Dis. 2017;11(7):e0005765–e. 10.1371/journal.pntd.0005765 28742087 PMC5542690

[42] Trent B, Liang Y, Xing Y, Esqueda M, Wei Y, Cho NH, et al Polarized lung inflammation and Tie2/angiopoietin-mediated endothelial dysfunction during severe *Orientia tsutsugamushi* infection. PLoS Negl Trop Dis. 2020;14(3):e0007675 10.1371/journal.pntd.0007675 32119672 PMC7067486

[43] Osterloh A, Papp S, Moderzynski K, Kuehl S, Richardt U, Fleischer B. Persisting *Rickettsia typhi* Causes Fatal Central Nervous System Inflammation. Infect Immun. 2016;84(5):1615–32. 10.1128/IAI.00034-16 26975992 PMC4862704

[44] Moderzynski K, Papp S, Rauch J, Heine L, Kuehl S, Richardt U, et al CD4+ T Cells Are as Protective as CD8+ T Cells against *Rickettsia typhi* Infection by Activating Macrophage Bactericidal Activity. PLoS Negl Trop Dis. 2016;10(11):e0005089. 27875529 10.1371/journal.pntd.0005089 PMC5119731

[45] Jiang L, Morris EK, Aguilera-Olvera R, Zhang Z, Chan T-C, Shashikumar S, et al Dissemination of *Orientia tsutsugamushi*, a causative agent of scrub typhus, and immunological responses in the humanized DRAGA mouse. Frontiers in immunology. 2018;9:816–. 10.3389/fimmu.2018.00816 29760694 PMC5936984

[46] Gong B, Lee YS, Lee I, Shelite TR, Kunkeaw N, Xu G, et al Compartmentalized, functional role of angiogenin during spotted fever group rickettsia-induced endothelial barrier dysfunction: evidence of possible mediation by host tRNA-derived small noncoding RNAs. BMC Infect Dis. 2013;13:285 10.1186/1471-2334-13-285 23800282 PMC3699377

[47] Weil A, Haymaker W. The distribution of the pathologic lesions of the central nervous system in scrub typhus (tsutsugamushi disease). J Neuropathol Exp Neurol. 1946;5:271–84. 10.1097/00005072-194610000-00001 21000555

[48] Sekeyová Z, Danchenko M, Filipčík P, Fournier PE. Rickettsial infections of the central nervous system. PLoS Negl Trop Dis. 2019;13(8):e0007469 10.1371/journal.pntd.0007469 31465452 PMC6715168

[49] Fang R, Ismail N, Walker DH. Contribution of NK cells to the innate phase of host protection against an intracellular bacterium targeting systemic endothelium. Am J Pathol. 2012;181(1):185–95. 10.1016/j.ajpath.2012.03.020 22617213 PMC3388147

[50] Woods ME, Olano JP. Host defenses to *Rickettsia rickettsii* infection contribute to increased microvascular permeability in human cerebral endothelial cells. J Clin Immunol. 2008;28(2):174–85. 10.1007/s10875-007-9140-9 17957455 PMC3691998

[51] Rydkina E, Turpin LC, Sahni SK. Rickettsia rickettsii infection of human macrovascular and microvascular endothelial cells reveals activation of both common and cell type-specific host response mechanisms. Infect Immun. 2010;78(6):2599–606. 10.1128/IAI.01335-09 20385756 PMC2876542

[52] Cho NH, Seong SY, Huh MS, Kim NH, Choi MS, Kim IS. Induction of the gene encoding macrophage chemoattractant protein 1 by *Orientia tsutsugamushi* in human endothelial cells involves activation of transcription factor activator protein 1. Infect Immun. 2002;70(9):4841–50. 10.1128/iai.70.9.4841-4850.2002 12183528 PMC128290

[53] Clifton DR, Rydkina E, Huyck H, Pryhuber G, Freeman RS, Silverman DJ, et al Expression and secretion of chemotactic cytokines IL-8 and MCP-1 by human endothelial cells after *Rickettsia rickettsii* infection: regulation by nuclear transcription factor NF-kappaB. Int J Med Microbiol. 2005;295(4):267–78. 10.1016/j.ijmm.2005.05.006 16128401

[54] Bohácsová M, Filipčík P, Opattová A, Valáriková J, Quevedo Diaz M, Škultéty L, et al Survival of rat cerebrocortical neurons after rickettsial infection. Microbes Infect. 2015;17(11–12):845–9. 10.1016/j.micinf.2015.09.024 26432946

[55] Joshi SG, Kovács AD. Rickettsia rickettsii infection causes apoptotic death of cultured cerebellar granule neurons. J Med Microbiol. 2007;56(Pt 1):138–41. 10.1099/jmm.0.46826-0 17172530

[56] Soong L. Dysregulated Th1 immune and vascular responses in scrub typhus pathogenesis. J Immunol. 2018;200(4):1233–40. 10.4049/jimmunol.1701219 29431689 PMC5812358

[57] Gharaibeh M, Hagedorn M, Lilla S, Hauptmann M, Heine H, Fleischer B, et al Toll-like receptor 2 recognizes *Orientia tsutsugamushi* and increases susceptibility to murine experimental scrub typhus. Infection and Immunity. 2016;84(12):3379–87. 10.1128/IAI.00185-16 27620720 PMC5116716

[58] Mendell NL, Bouyer DH, Walker DH. Murine models of scrub typhus associated with host control of *Orientia tsutsugamushi* infection. PLoS Negl Trop Dis. 2017;11(3):e0005453–e. 10.1371/journal.pntd.0005453 28282373 PMC5362142

[59] Astrup E, Janardhanan J, Otterdal K, Ueland T, Prakash JA, Lekva T, et al Cytokine network in scrub typhus: high levels of interleukin-8 are associated with disease severity and mortality. PLoS Negl Trop Dis. 2014;8(2):e2648 10.1371/journal.pntd.0002648 24516677 PMC3916254

[60] Otterdal K, Janardhanan J, Astrup E, Ueland T, Prakash JAJ, Lekva T, et al Increased endothelial and macrophage markers are associated with disease severity and mortality in scrub typhus. Journal of Infection. 2014;69(5):462–9. 10.1016/j.jinf.2014.06.018 24995849

[61] Walker DH, Dumler JS. Emerging and reemerging rickettsial diseases. N Engl J Med. 1994;331(24):1651–2. 10.1056/NEJM199412153312410 7969347

[62] Moron CG, Popov VL, Feng HM, Wear D, Walker DH. Identification of the target cells of *Orientia tsutsugamushi* in human cases of scrub typhus. Mod Pathol. 2001;14(8):752–9. 10.1038/modpathol.3880385 11504834

[63] Osterloh A. Immune response against rickettsiae: lessons from murine infection models. Med Microbiol Immunol. 2017;206(6):403–17. 10.1007/s00430-017-0514-1 28770333 PMC5664416

[64] Soong L, Wang H, Shelite TR, Liang Y, Mendell NL, Sun J, et al Strong type 1, but impaired type 2, immune responses contribute to *Orientia tsutsugamushi*-induced pathology in mice. PLoS Negl Trop Dis. 2014;8(9):e3191 10.1371/journal.pntd.0003191 25254971 PMC4177881

